# Comparison of safety of general anesthesia and intravenous sedation during third-molar extraction surgery

**DOI:** 10.1038/s41598-024-67045-2

**Published:** 2024-07-19

**Authors:** Se-Ung Park, Taewoo Kim, Jiwon Do, Mincheul Cho, Jung-Sub An, Myong-Hwan Karm

**Affiliations:** 1 Department of Anesthesiology and Pain Medicine, Kyung Hee University Hospital at Gangdong, Seoul, 05278 Republic of Korea; 2 Department of Dental Anesthesiology, School of Dentistry and Dental Research Institute, Seoul National University, 101 Daehak-Ro, Jongno-Gu, Seoul, 03080 Republic of Korea; 3 Department of Oral and Maxillofacial Surgery, Seoul National University Dental Hospital, Seoul, 03080 Republic of Korea; 4 Department of Anesthesiology and Pain Medicine, Seoul National University Bundang Hospital, Seoul, 13620 Republic of Korea; 5 Department of Orthodontics, School of Dentistry and Dental Research Institute, Seoul National University, 101 Daehak-Ro, Jongno-Gu, Seoul, 03080 Republic of Korea

**Keywords:** Anesthesia, general, Anesthesia, intravenous, Conscious sedation, Dexmedetomidine, Safety, Health care, Medical research

## Abstract

This study compared the safety of general anesthesia (GA) and intravenous sedation (IVS) in patients who underwent extraction of one or more third molars. Data from 1260 patients (GA group, n = 1043; IVS group, n = 217) were retrospectively analyzed, including demographics, preoperative data, intraoperative hemodynamic parameters (blood pressure, heart rate, and oxygen saturation level), and medications administered intraoperatively and postoperatively. The incidence of intraoperative circulatory variations, surgery and anesthesia durations, postoperative complications, and medication use were assessed and compared. The GA group had longer anesthesia and surgery durations, a higher incidence of hypotension, and a higher frequency of postoperative analgesic use than the IVS group. Dexmedetomidine was the most frequently used sedative agent. The IVS group had a lower incidence of intraoperative hypotension but they had a higher need for vasopressors in the recovery room. Both anesthesia methods maintained satisfactory oxygen saturation levels and sufficient anesthesia throughout the procedure, but they showed different characteristics regarding the duration of surgery and anesthesia duration, hemodynamic stability, and postoperative analgesic needs. IVS may be preferable for patients at risk of cardiovascular complications such as hypotension or tachycardia during surgery.

## Introduction

Extraction of the third molar is the most frequently performed procedure in oral and maxillofacial surgery^1^. Owing to insufficient space between the anterior border of the mandibular ramus and the distal surface of the second molar as well as anatomical factors such as palatal impaction, the mandibular third molar has a high impaction rate^2^. In relation to maxillary third molars, factors such as proximity to the maxillary sinus and the presence of dense cortical bone can contribute to impaction of the third molar^3^. The procedure, commonly performed under local anesthesia, is increasingly being done under general anesthesia (GA) or intravenous sedation (IVS) to address patients’ anxiety, fear, and stress regarding potential pain. This trend is in line with the emphasis on the quality of life, leading to a notable increase in the number of patients opting for GA or IVS^4^. Patients with more complex cases or with anticipated complications require an additional sedation method^5^.

The choice of anesthesia method and sedation level depends on factors such as patient's medical history, procedure complexity, anxiety level, surgery and anesthesia duration, and number of teeth to be extracted^4,6^. GA involves a complete lack of response to any stimulus, requiring support for airway management and influencing both respiratory and cardiovascular functions. The IVS maintains a purposeful response to verbal communication, with less demand for airway management compared with GA and with minimal impact on respiratory and cardiovascular functions. IVS can range from moderate, in which the patient responds to normal speech without any impact on respiratory or cardiac function, to deep sedation, in which the patient may respond only to repeated painful stimuli and may need assistance in keeping the airway open^7^. Both GA and IVS require careful monitoring of hemodynamic changes and intensive patient respiratory monitoring. Observation of adverse reactions and appropriate interventions should be performed not only during the surgical procedure but also during recovery^8,9^.

Although the use of GA and IVS for third-molar extraction is gradually increasing, research on the differences in the characteristics of these two anesthesia methods is limited. The aim of this study was to investigate the differences in anesthesia characteristics, including the perioperative statistics and risk of intraoperative complications, among patients who underwent third-molar extraction with GA and IVS.

## Results

**Figure 1:**
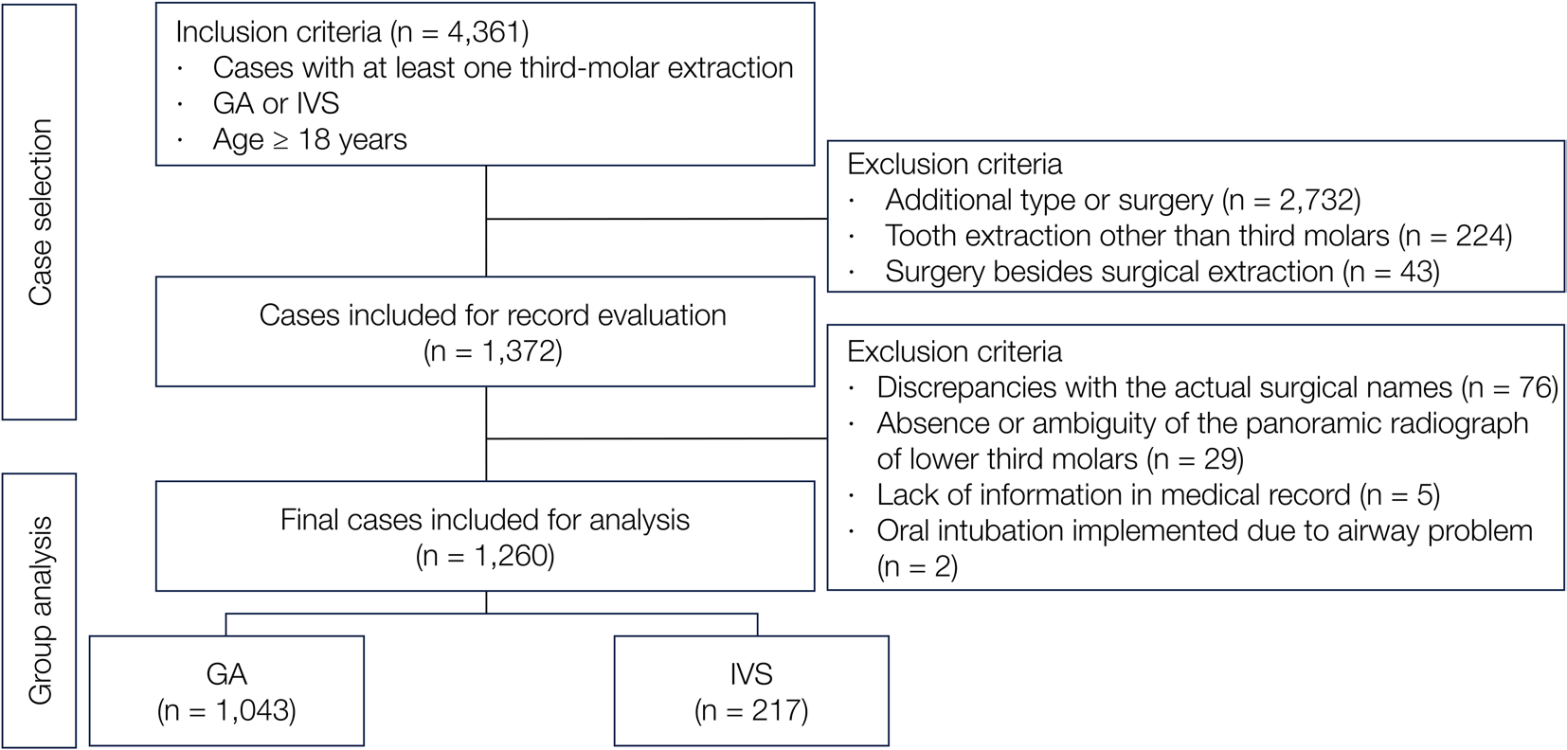
Flowchart of patient selection. GA, general anesthesia; IVS, intravenous sedation.

A total of 4361 patients underwent extraction of one or more third molars using GA or IVS. Data from 3101 patients were excluded according to the exclusion criteria; finally, data from 1260 patients were analyzed. Of these, 1043 underwent extraction under GA, whereas 217 underwent extraction with IVS (Fig. 1).

The GA and IVS groups were similar in terms of age, sex, height, weight, body mass index (BMI), underlying diseases, history of surgery, allergy history, and preoperative systolic and diastolic blood pressure (BP) (Table 1). Significant differences in the preoperative American Society of Anesthesiologists (ASA) classification distribution and heart rate (HR) were observed between the two groups. The IVS group showed a more severe distribution of ASA classification than the GA group (P = 0.001). Meanwhile, the GA group had a higher HR (80.42 ± 13.17 bpm) than the IVS group (77.75 ± 12.98 bpm) (P = 0.007). Patients in the GA group had higher preoperative temperature (36.69 °C ± 0.35 °C) than the IVS group (36.63 °C ± 0.31 °C, P = 0.019) (Table 1). In the comparison of anesthesia methods between the GA and IVS groups, the GA method primarily used inhalation agents in 823 cases (79%) and total intravenous anesthesia (TIVA) in 220 cases (21%). For the IVS method, dexmedetomidine was the predominant agent used in 208 cases (95.85%), whereas other agents such as propofol, diazepam, remifentanil, and midazolam were used in minor cases. No anesthetic failures occurred in the IVS group that required GA conversion.

**Table 1:** Demographic and clinical characteristics of patients undergoing third molar extraction.

Characteristics	Overall (n = 1260)	General anesthesia (n = 1043)	Intravenous sedation (n = 217)	*P*-value^†^
Age (year)	28.77 ± 9.76	28.69 ± 9.58	29.18 ± 10.59	0.495
Sex (male)	569 (45.16%)	475 (45.54%)	94 (43.32%)	0.549
Height (cm)	166.98 ± 8.56	167.13 ± 8.67	166.30 ± 8.00	0.199
Weight (kg)	63.79 ± 14.08	63.67 ± 14.29	64.37 ± 13.06	0.509
BMI (kg/m^2^)	22.73 ± 3.98	22.65 ± 4.03	23.13 ± 3.67	0.102
ASA status classification				
I	1012 (80.32%)	857 (82.17%)	155 (71.43%)	
II	238 (18.89%)	179 (17.16%)	59 (27.19%)	0.001
III	10 (0.79%)	7 (0.67%)	3 (1.38%)	
Underlying disease*	201 (15.95%)	160 (15.34%)	41 (18.89%)	0.193
History of surgery^‡^	472 (37.46%)	389 (37.30%)	83 (38.25%)	0.792
Allergy history	175 (13.89%)	147 (14.09%)	28 (12.90%)	0.644
Preoperative vital signs				
Systolic BP (mmHg)	121.06 ± 16.12	120.91 ± 15.97	121.76 ± 16.84	0.477
Diastolic BP (mmHg)	80.25 ± 11.98	80.09 ± 11.78	81.02 ± 12.92	0.330
HR (/min)	79.96 ± 13.17	80.42 ± 13.17	77.75 ± 12.98	0.007
Temp (°C)	36.68 ± 0.34	36.69 ± 0.35	36.63 ± 0.31	0.019

Data are presented as numbers (%) or means ± standard deviations.

*ASA* American Society of Anesthesiologists, *BMI* body mass index, *BP* blood pressure, *HR* heart rate, *n* number, *Temp* temperature.

*Underlying diseases include cardiovascular, pulmonological, hematological, endocrine, hepatological, psychological, genetic, neurological diseases, and allergies.

^‡^History of surgery includes orthognathic, orofacial, chest, abdominal, limb, spine surgery, and surgeries related to facial cleft.

^†^Independent t-test was performed for continuous variables, and Fisher's exact test was performed for categorical variables.

The location and frequency of extracted third molars in the two groups are shown in Table 2. The number and location of extracted teeth were comparable between the two groups. In both groups, third-molar extraction was the most frequently performed surgery (GA group, 50.91%; IVS group, 43.78%), followed by bilateral extraction of two mandibular third molars (GA group, 18.60%; IVS group, 20.28%) (Table 2).

**Table 2:** The location and frequency of extracted third molars in the two groups.

Extracted tooth	Overall (n = 1260)	General anesthesia (n = 1043)	Intravenous sedation (n = 217)	*P*-value^†^
#18	12 (0.95%)	10 (0.96%)	2 (0.92%)	0.134
#28	9 (0.71%)	8 (0.77%)	1 (0.46%)	
#38	46 (3.65%)	35 (3.36%)	11 (5.07%)	
#48	50 (3.97%)	36 (3.45%)	14 (6.45%)	
#18, #28	13 (1.03%)	11 (1.05%)	2 (0.92%)	
#18, #38	8 (0.63%)	7 (0.67%)	1 (0.46%)	
#18, #48	21 (1.67%)	18 (1.73%)	3 (1.38%)	
#28, #38	16 (1.27%)	12 (1.15%)	4 (1.84%)	
#28, #48	5 (0.40%)	2 (0.19%)	3 (1.38%)	
#38, #48	238 (18.89%)	194 (18.60%)	44 (20.28%)	
#18, #28, #38	28 (2.22%)	24 (2.30%)	4 (1.84%)	
#18, #28, #48	20 (1.59%)	18 (1.73%)	2 (0.92%)	
#18, #38, #48	78 (6.19%)	59 (5.66%)	19 (8.76%)	
#28, #38, #48	90 (7.14%)	78 (7.48%)	12 (5.53%)	
#18, #28, #38, #48	626 (49.68%)	531 (50.91%)	95 (43.78%)	

Data are presented as numbers (%).

^†^Fisher's exact test was performed.

^#^18, maxillary right third molar; #28, maxillary left third molar; #38, mandibular left third molar; #48 mandibular right third molar.

Table 3 presents the reasons and frequency of third-molar extraction according to location in the two groups. Prophylactic removal was the most common reason for extraction, followed by pericoronitis, orthodontic treatment, dental caries, and pathological effects on adjacent teeth (Table 3).

**Table 3:** Reasons and frequency of third molar extraction according to location.

	Causes	Overall	General anesthesia	Intravenous sedation
#18	Prophylactic	669 (83.00%)	559 (82.45%)	110 (85.94%)
	Pericoronitis	26 (3.23%)	24 (3.54%)	2 (1.56%)
	Dental caries	28 (3.47%)	23 (3.39%)	5 (3.91%)
	Orthodontic	72 (8.93%)	64 (9.44%)	8 (6.25%)
	Pathological effects on adjacent teeth	10 (1.24%)	7 (1.03%)	3 (2.34%)
	Others	1 (0.12%)	1 (0.15%)	0 (0%)
	Subtotal	806	678	128
#28	Prophylactic	667 (82.65%)	563 (82.31%)	104 (84.55%)
	Pericoronitis	27 (3.35%)	22 (3.22%)	5 (4.07%)
	Dental caries	34 (4.21%)	27 (3.95%)	7 (5.69%)
	Orthodontic	68 (8.43%)	62 (9.06%)	6 (4.88%)
	Pathological effects on adjacent teeth	11 (1.36%)	10 (1.46%)	1 (0.81%)
	Others	0 (0%)	0 (0%)	0 (0%)
	Subtotal	807	684	123
#38	Prophylactic	701 (62.04%)	588 (62.55%)	113 (59.47%)
	Pericoronitis	275 (24.34%)	219 (23.30%)	56 (29.47%)
	Dental caries	29 (2.57%)	22 (2.34%)	7 (3.68%)
	Orthodontic	80 (7.08%)	72 (7.66%)	8 (4.21%)
	Pathological effects on adjacent teeth	26 (2.30%)	23 (2.45%)	3 (1.58%)
	Others	19 (1.68%)	16 (1.7%)	3 (1.58%)
	Subtotal	1130	940	190
#48	Prophylactic	688 (60.99%)	578 (61.75%)	110 (57.29%)
	Pericoronitis	272 (24.11%)	216 (23.08%)	56 (29.17%)
	Dental caries	31 (2.75%)	24 (2.56%)	7 (3.65%)
	Orthodontic	84 (7.45%)	76 (8.12%)	8 (4.17%)
	Pathological effects on adjacent teeth	22 (1.95%)	19 (2.03%)	3 (1.56%)
	Others	31 (2.75%)	23 (2.46%)	8 (4.17%)
	Subtotal	1128	936	192
Total	Prophylactic	2725 (70.40%)	2288 (70.66%)	437 (69.04%)
	Pericoronitis	600 (15.50%)	481 (14.85%)	119 (18.8%)
	Dental caries	122 (3.15%)	96 (2.96%)	26 (4.11%)
	Orthodontic	304 (7.85%)	274 (8.46%)	30 (4.74%)
	Pathological effects on adjacent teeth	69 (1.78%)	59 (1.82%)	10 (1.58%)
	Others	51 (1.32%)	40 (1.24%)	11 (1.74%)
	Subtotal	3871	3238	633

Duplicate entries are allowed.

^#^18, maxillary right third molar; #28, maxillary left third molar; #38, mandibular left third molar; #48 mandibular right third molar.

The preoperative laboratory results of the two groups are shown in Table 4. There were no significant differences between the two groups in most preoperative laboratory test results. However, the activated partial thromboplastin time and neutrophil and alkaline phosphatase levels were significantly greater in the GA group than in the IVS group (P < 0.05), whereas albumin levels were greater in the IVS group than in the GA group (P = 0.004) (Table 4).

**Table 4:** Preoperative laboratory results of the two groups.

Parameters	Overall (n = 1260)	General anesthesia (n = 1043)	Intravenous sedation (n = 217)	*P*-value^†^
Hemoglobin (g/dL)	14.23 ± 1.49	14.26 ± 1.46	14.08 ± 1.65	0.104
Hematocrit (%)	42.13 ± 3.83	42.21 ± 3.77	41.77 ± 4.07	0.126
Platelet count (× 10^9^/L)	259.72 ± 55.51	259.42 ± 56.11	261.18 ± 52.63	0.670
aPTT (seconds)	35.74 ± 4.25	35.95 ± 4.13	34.71 ± 4.68	< 0.001
INR	1.01 ± 0.09	1.01 ± 0.08	1.02 ± 0.11	0.290
PT (seconds)	99.27 ± 10.52	99.36 ± 10.43	98.85 ± 10.95	0.523
Neutrophil (cells/μL)	3476.79 ± 1270.31	3515.14 ± 1314.25	3292.45 ± 1015.93	0.006
ESR (mm/h)	9.15 ± 9.17	9.11 ± 9.00	9.38 ± 10.01	0.691
Calcium (mg/dL)	9.35 ± 0.37	9.35 ± 0.38	9.32 ± 0.31	0.250
Phosphorus (mg/dL)	3.59 ± 0.45	3.6 ± 0.46	3.56 ± 0.43	0.177
Glucose (mg/dL)	93.21 ± 11.67	92.97 ± 12.00	94.37 ± 9.88	0.106
BUN (mg/dL)	11.79 ± 3.16	11.76 ± 3.14	11.92 ± 3.29	0.502
Uric acid (mg/dL)	5.23 ± 1.44	5.26 ± 1.46	5.09 ± 1.31	0.103
Cholesterol (mg/dL)	189.13 ± 29.22	189.13 ± 30.45	189.11 ± 22.44	0.989
Total protein (g/dL)	7.43 ± 0.38	7.44 ± 0.38	7.33 ± 0.36	< 0.001
Albumin (g/dL)	4.54 ± 0.29	4.53 ± 0.29	4.59 ± 0.27	0.004
Total bilirubin (mg/dL)	0.84 ± 0.50	0.83 ± 0.47	0.90 ± 0.59	0.101
Alkaline phosphatase (IU/L)	42.45 ± 34.17	44.17 ± 31.53	34.14 ± 43.88	0.002
Aspartate transaminase (IU/L)	21.97 ± 11.14	21.82 ± 10.62	22.68 ± 13.36	0.300
Alanine transaminase (IU/L)	23.18 ± 21.04	22.92 ± 21.28	24.40 ± 19.85	0.348
Creatinine (mg/dL)	0.84 ± 0.16	0.84 ± 0.16	0.83 ± 0.15	0.324
Sodium (mEq/L)	140.91 ± 1.73	140.95 ± 1.73	140.76 ± 1.72	0.151
Potassium (mEq/L)	4.29 ± 0.32	4.29 ± 0.32	4.30 ± 0.33	0.713
Chloride (mEq/L)	103.65 ± 1.77	103.61 ± 1.77	103.85 ± 1.75	0.077

*aPTT* activated partial thromboplastin time, *INR* international normalized ratio, *PT* prothrombin time, *ESR* erythrocyte sedimentation rate, *BUN* blood urea nitrogen, *AST* aspartate aminotransferase, *ALT* alanine aminotransferase.

^†^Independent t-test was performed.

The intraoperative and postoperative characteristics of the two groups are presented in Table 5. The durations of both anesthesia and surgery were significantly longer in the GA group than in the IVS group. The patients in the GA group underwent 60.36 min of surgery and 91.16 min of anesthesia, whereas those in the IVS group underwent 49.82 min of surgery and 70.85 min of anesthesia (P < 0.001). Total fluid volume and total blood loss were also higher in the GA group than in the IVS group (P < 0.001). Vital signs showed that the GA group had lower mean systolic, diastolic, and mean BPs, with more frequent episodes of hypotension (GA group, 21.57%; IVS group, 8.29%) and tachycardia (GA group, 9.30%; IVS group, 0.46%) (P < 0.001). The IVS group had a higher incidence of hypertension (IVS group, 4.15%; GA group, 1.44%) (P = 0.008) and bradycardia (IVS group, 33.18%; GA group, 18.12%) (P < 0.001). Average oxygen saturation was significantly higher in IVS (99.2%) than in GA (98.7%) (P < 0.001). Minimum saturation was maintained at > 90% in all patients. The proportion of patients who received opioids (199 cases, 19.08%) and nonsteroidal anti-inflammatory drugs (NSAIDs) (78 cases, 7.48%) during the recovery period was higher in the GA group than in the IVS group (opioids, 3 cases, 1.38%; NSAIDs, 1 case, 0.46%) (P < 0.001). Anti-emetic use was more frequent in the GA group (7 cases) than in the IVS group (0 case), but the difference was not statistically significant. Vasopressors were used exclusively in the IVS group (20 cases, 9.22%, P < 0.001), and there was no significant difference in the use of antihypertensives between the two groups. None of the patients experienced severe adverse events following surgery.

**Table 5:** Intraoperative and postoperative characteristics of patients undergoing third molar extraction in the two groups.

Parameters	Overall (n = 1260)	General anesthesia (n = 1043)	Intravenous sedation (n = 217)	*P*-value^†^
Intraoperative statistics				
Duration of surgery (min)	58.55 ± 31.85	60.36 ± 33.07	49.82 ± 23.33	< 0.001
Duration of anesthesia (min)	87.66 ± 33.43	91.16 ± 33.97	70.85 ± 24.57	< 0.001
Total fluid volume (ml)	270.56 ± 155.25	286.63 ± 160.89	193.32 ± 91.48	< 0.001
Total blood loss (ml)	46.88 ± 62.50	51.56 ± 66.03	24.38 ± 33.40	< 0.001
BP				
Systolic BP (mmHg)	109.1 ± 13.49	107.6 ± 12.96	116.28 ± 13.72	< 0.001
Diastolic BP (mmHg)	57.23 ± 9.52	56.42 ± 9.34	61.13 ± 9.44	< 0.001
Mean BP (mmHg)	78.55 ± 9.80	77.63 ± 9.53	82.96 ± 9.89	< 0.001
Episodes of hypotension	243 (19.29%)	225 (21.57%)	18 (8.29%)	< 0.001
Ephedrine	13 (1.03%)	13 (1.25%)	0 (0%)	0.098
Episodes of hypertension	24 (1.90%)	15 (1.44%)	9 (4.15%)	0.008
HR				
HR (beats/min)	79.54 ± 12.91	80.61 ± 12.97	74.39 ± 11.28	< 0.001
Episodes of bradycardia	261 (20.71%)	189 (18.12%)	72 (33.18%)	< 0.001
Atropine or glycopyrrolate	8 (0.63%)	6 (0.58%)	2 (0.92%)	0.559
Episodes of tachycardia	98 (7.78%)	97 (9.30%)	1 (0.46%)	< 0.001
Esmolol	5 (0.4%)	5 (0.48%)	0 (0%)	0.307
Saturation				
Saturation (%)	98.79 ± 0.89	98.70 ± 0.89	99.20 ± 0.76	< 0.001
Saturation < 95%	12 (0.95%)	10 (0.96%)	2 (0.92%)	0.959
Saturation < 90%	0 (0%)	0 (0%)	0 (0%)	
Medication during recovery				
Opioid	202 (16.03%)	199 (19.08%)	3 (1.38%)	< 0.001
NSAIDs	79 (6.27%)	78 (7.48%)	1 (0.46%)	< 0.001
Antiemetics	7 (0.56%)	7 (0.67%)	0 (0%)	0.226
Vasopressor	20 (1.59%)	0 (0%)	20 (9.22%)	< 0.001
Anti-hypertensive	4 (0.32%)	4 (0.38%)	0 (0%)	0.361

Data are presented as numbers (%) or means ± standard deviations.

Episode of hypotension, systolic BP < 80% baseline; episodes of hypertension, systolic BP > 120% baseline; episodes of bradycardia, HR < 60/min; episodes of tachycardia, HR > 120/min.

*BP* blood pressure, *HR* heart rate, *NSAIDs* non-steroidal anti-inflammatory drugs.

^†^Independent t-test was performed for continuous variables, and Fisher's exact test was performed for categorical variables.

## Discussion

The present study aimed to investigate the differences in anesthesia characteristics between patients who underwent third-molar extraction with GA and IVS. When comparing the two groups, patients under IVS showed a significantly shorter surgical and anesthesia durations than those under GA. The longer operation time in the GA group could be explained by the lack of consciousness to cooperate with mouth opening and positioning^10^. The duration of anesthesia was longer in the GA group than in the IVS group, mainly because of the additional time required for intubation, extubation, and waking up^11,12^. A shorter duration implies increased turnover, efficiency, and cost effectiveness.

Intraoperative hypertension and bradycardia, postoperative hypotension requiring vasopressors, and less usage of pain control were observed in patients receiving IVS, which was mostly performed using dexmedetomidine for sedation. Dexmedetomidine functions as an agonist of α2-adrenergic receptors and exhibits sympatholytic properties by lowering the HR and BP^13^. Dexmedetomidine has an anesthetic-sparing effect^14–16^. A rapid bolus infusion of dexmedetomidine results in elevated arterial pressure^17^. These properties of dexmedetomidine could explain the initial increase in BP, the sympatholytic effect leading to postoperative hypotension in the recovery room with the increased use of vasopressors, and the reduction in analgesic demand in the postoperative recovery room.

The average systolic, diastolic, and mean BP were lower in the GA group than in the IVS group, and the incidence of a 20% decrease from baseline systolic BP was significantly higher. The inhalation and intravenous agents used in GA contribute to a decrease in myocardial contractility and induce vasodilation, thereby lowering the BP^18,19^. Cases of hypertension, defined as a 20% increase from the baseline systolic BP, were more frequent in the IVS group. This could be attributed to anxiety and pain associated with conscious patients undergoing surgery^20^. Therefore, it would be safer to choose the IVS over the GA in patients who may experience cardiovascular complications such as hypotension during third-molar extraction^4^. Intraoperative saturation levels were significantly lower in the GA group (average, 98.7%) than in the IVS group, but the difference was not clinically significant. The frequency of saturation levels dropping below 95% occurred at a similar rate in both groups, and there were no instances of hypoxemia in either group where levels fell below 90%. Studies have indicated that hypoxemia frequently occurs during surgery performed under IVS^13^. However, the patients in our study predominantly received dexmedetomidine for sedation, which has the advantage of fewer respiratory complications^21^. Thus, sedatives other than dexmedetomidine requires additional attention to the possibility of respiratory complications.

The use of analgesics such as opioids or NSAIDs in the recovery room was significantly higher in the GA group. This could be explained by the additional pain from nasal intubation and extubation required in GA^22^ and the analgesic effect of dexmedetomidine used in IVS^14^. The difference in the use of vasopressors in the recovery rooms could be connected to hypotension caused by the sympatholytic action of dexmedetomidine^17,23^, and in the adverse effect of the drug necessitates caution.

Owing to the retrospective nature of this study, there are several limitations. There was an uneven distribution of patients in the GA and IVS groups. Patient selection could be biased by the surgical difficulty and patient condition, which might influence the choice of anesthesia method and affect the outcomes. Despite using standardized anesthesia and surgical protocols, the variability among surgeons and anesthesiologists could also affect the outcomes. The total dosage of the local anesthetic and the site for local anesthesia, which could interfere with the postoperative usage of analgesics, were not assessed. The use of 5-min interval records for intraoperative vital sign monitoring may have missed instances exceeding specific thresholds. Complications, such as vomiting, prolonged recovery, and new cardiac dysrhythmias, were not evaluated. There has been no analysis on the impact of the difficulty of extraction, which could influence the duration of surgery, total fluid volume, and total blood loss. Further studies on this topic will be conducted. Nevertheless, the inclusion of 1260 patients in this study provides valuable insights into patient conditions during surgery and the medications used during surgery and in the recovery room. Furthermore, to our knowledge, this study represents the first comparison between GA and IVS in third-molar extraction. Further research is needed to compare GA and IVS after adjusting for differences related to anesthesia and procedural difficulties.

In conclusion, this study demonstrated that both GA and IVS can be safely administered for third-molar extraction. For patients who are anticipated to experience cardiovascular events, such as hypotension or tachycardia, during surgery, we suggest IVS as anesthesia. Information on the choice of anesthetics for GA and IVS and interventions used for the control of intraoperative events would aid in the selection of anesthetic methods for various dental surgeries beyond third-molar extractions. Additional prospective studies related to the efficacy and safety of GA and IVS will be needed in the future.

## Methods

The study design was approved by the Institutional Review Board of Seoul National University School of Dentistry (approval number S-D20220015), and all research was performed in accordance with relevant guidelines and the Declaration of Helsinki. The requirement for informed consent was waived owing to the retrospective nature of the study using the electronic medical records (EMRs).

### Study design, sample, and data collection

This study analyzed the EMRs of patients who underwent at least a one third-molar extraction under the care of the Department of Dental Anesthesiology, with procedures performed by the Oral and Maxillofacial Surgery Department at Seoul National University Dental Hospital from 2015 to 2021. The study included patients receiving GA or IVS; those who underwent additional surgeries or had extractions of teeth other than the third molars were excluded.

Data collection involved a thorough chart review to gather comprehensive information, including demographic details (age, sex, body weight, height, and BMI); medical, surgical, and allergy history; preoperative vital signs; and the ASA physical status classification. The number of third molars removed and the rationale for each extraction were documented. Preoperative assessments included laboratory examinations, panoramic dental radiographs, chest X-rays, and 12-lead electrocardiography (ECGs). Intraoperative data included the duration of surgery and anesthesia, along with the types and dosages of drugs and fluids administered. Postoperative evaluations in the recovery room recorded the BP (systolic, diastolic, and mean), HR, peripheral oxygen saturation (SpO_2_), end-tidal carbon dioxide, and body temperature at 5-min intervals, excluding the induction and emergence phases.

The primary outcome assessed was the incidence of intraoperative circulatory variations such as hypotension, hypertension, bradycardia, tachycardia, and hypoxemia, defined by specific criteria, including changes in BP, HR, and SpO_2_ levels. Intraoperative hypotension was defined as a decrease in preoperative systolic BP to < 80% of the baseline value or the use of a vasopressor such as ephedrine^24^. Hypertension was defined as an average systolic BP exceeding 20% of the preoperative baseline or the use of antihypertensive medication^25^. Bradycardia was defined as a 5-min average HR decreasing to < 60 bpm or the administration of anticholinergic medications, such as atropine or glycopyrrolate^26^. Tachycardia was defined as an average HR > 120 bpm or the use of esmolol to decrease HR^27^. Hypoxemia was defined as SpO_2_ < 95%, and severe hypoxemia as SpO_2_ < 90%^28^. The duration of anesthesia or surgery and records of other anesthetic complications were evaluated.

### Anesthetic techniques

Anesthetic management was conducted by experienced anesthesiologists with more than 5 years of experience under a unified set of policies and protocols for anesthesia^29^. All patients underwent standard preoperative assessments, including a review of their medical history, measurement of vital signs, routine laboratory examinations, ECG, and chest posteroanterior radiography. All assessments were conducted within 3 months before the surgical procedure. No pre-anesthetic medications were administered before the patients arrived in the operating room.

For patients undergoing GA, standard monitoring included pulse oximetry, ECG, non-invasive BP, end-tidal carbon dioxide, and bispectral index (BIS). GA was initiated with the administration of an induction agent (propofol, thiopental, or etomidate) and neuromuscular-blocking agents (rocuronium or vecuronium), followed by maintenance with a volatile agent (sevoflurane or desflurane) or TIVA using propofol and remifentanil through a nasotracheal tube. Anesthesia was carefully adjusted to maintain a BIS between 40 and 60. Systolic BP was maintained between 90 and 150 mmHg, and HR was maintained between 50 and 100 bpm, with anesthetic dose control and use of other medications according to hemodynamic change: atropine or glycopyrrolate was used for bradycardia; esmolol was used for tachycardia; ephedrine was used for hypotension. Following surgery, extubation was performed once consciousness and neuromuscular function had adequately recovered.

Patients with IVS were monitored using pulse oximetry, ECG, non-invasive BP, and end-tidal carbon dioxide, excluding BIS monitoring. Sedation was achieved using medications, such as dexmedetomidine, midazolam, propofol, remifentanil, or diazepam, with oxygen provided at a rate of 5 L/min through a nasal cannula. The choice of sedative was tailored to each patient’s need to maintain a controlled sedation level and ensure that vital signs remained within safe parameters. The vital signs of patients who underwent IVS were maintained at ranges similar to those of the patients in the GA group. Recovery from IVS involved regaining consciousness under close observation to ensure safety and comfort.

After surgery, the patients in both groups were observed in the recovery room to control postoperative complications. Opioids or NSAIDs were used for pain management. Ramosetron, an anti-emetic, was administered to treat nausea and vomiting. Patients with hypotension were given ephedrine to increase their BP, whereas those with hypertension were given nicardipine, labetalol, or hydralazine.

### Surgical techniques

All extractions were performed by oral and maxillofacial surgeons with several years of experience and with the same surgical policy and protocol. Local anesthetics (2% lidocaine with 1:100,000 epinephrine) were used for the infiltration anesthesia of the mucosa around the third-molar area and block anesthesia of the posterior superior alveolar nerve or inferior alveolar nerve. A full-thickness mucoperiosteal flap was created on the buccal mucosa of the third-molar area. Lateral subperiosteal dissection was performed to expose the tooth. A dental handpiece was used to remove the bone around the tooth. The tooth was extracted using tooth elevators or forceps. The sockets were curetted using a surgical curette and irrigated with saline solution. The flap created on the posterior maxilla or mandible was positioned back to its original location and sutured.

### Statistical analysis

Descriptive statistics are expressed as numbers (%) or means ± standard deviations. To analyze the differences in demographic, clinical, and aesthetic characteristics between the GA and IVS groups, independent t-test was performed for continuous variables and Fisher's exact test was performed for categorical variables. All statistics were performed using SPSS Statistics version 25 (IBM Corp., Armonk, NY, USA), and the level of significance was set at α = 0.05.

## Data Availability

The datasets generated during and/or analysed during the current study are available from the corresponding author on reasonable request.
